# Reporting and methodological quality of studies that use Mendelian randomisation in UK Biobank: a meta-epidemiological study

**DOI:** 10.1136/bmjebm-2022-112006

**Published:** 2022-12-08

**Authors:** Mark J Gibson, Francesca Spiga, Amy Campbell, Jasmine N Khouja, Rebecca C Richmond, Marcus R Munafò

**Affiliations:** 1 MRC Integrative Epidemiology Unit (IEU), University of Bristol, Bristol, UK; 2 School of Psychological Science, University of Bristol, Bristol, UK; 3 Bristol Medical School: Population Health Sciences, University of Bristol, Bristol, UK

**Keywords:** Epidemiology, Public health

## Abstract

**Objectives:**

To identify whether Mendelian randomisation (MR) studies are appropriately conducted and reported in enough detail for other researchers to accurately replicate and interpret them.

**Design:**

Cross-sectional meta-epidemiological study.

**Data sources:**

Web of Science, EMBASE, PubMed and PsycINFO were searched on 15 July 2022 for literature.

**Eligibility criteria:**

Full research articles that conducted an MR analysis exclusively using individual-level UK Biobank data to obtain a causal estimate of the exposure–outcome relationship (for no more than ten exposures or outcomes).

**Methods and analysis:**

Data were extracted using a 25-item checklist relating to reporting and methodological quality (based on the Strengthening the Reporting of Observational Studies in Epidemiology (STROBE)-MR reporting guidelines and the guidelines for performing MR investigations). Article characteristics, such as 2021 Journal Impact Factor, publication year, journal word limit/recommendation, whether the MR analysis was the primary analysis, open access status and whether reporting guidelines were followed, were also extracted. Descriptive statistics were calculated for each item, and whether article characteristics predicted overall article completeness was investigated with linear regression.

**Results:**

116 articles were included in this review. The proportion of articles which reported complete information/adequate methodology ranged from 3% to 100% across the different items. Palindromic variants, variant replication, missing data, associations of the instrumental variable with the exposure or outcome and bias introduced by two-sample methods used on a single sample were often not completely addressed (<11%). There was no clear evidence that article characteristics predicted overall completeness except for primary analysis status.

**Conclusions:**

The results identify areas in which the reporting and conducting of MR studies needs to be improved and also suggest researchers do not make use of supplementary materials to sufficiently report secondary analyses. Future research should focus on the quality of code and analyses, attempt direct replications and investigate the impact of the STROBE-MR specifically.

**Study registration:**

https://osf.io/nwrdj

**WHAT IS ALREADY KNOWN ON THIS TOPIC**

- Mendelian randomisation (MR) is becoming more widely used each year and, while recently created reporting and methodological guidelines exist, little is known about the reporting or methodological quality of published MR articles.
- Previous systematic reviews suggest reporting quality is poor in certain areas, but these reviews focus on a narrow range of reporting outcomes and articles.

**WHAT THIS STUDY ADDS**

- This study found that, across a sample of 116 MR articles from all fields, and across a broad range of data-extraction items related to reporting and methodological quality (partly based on the Strengthening the Reporting of Observational Studies in Epidemiology-MR reporting guidelines), quality varied greatly and was particularly poor for several items.
- This study also found that factors such as Journal Impact Factor, year of publication and journal word limit/recommendations did not clearly predict overall article completeness.

**HOW THIS STUDY MIGHT AFFECT RESEARCH, PRACTICE OR POLICY**

- This study highlights that the reporting and methodological quality of MR articles needs to be improved as, without methodologically sound analyses and transparent reporting, results cannot be adequately interpreted, and therefore the impact of the research and the benefit gained from public funding is reduced.

## Introduction

Mendelian randomisation (MR) is a method of causal inference that uses genetic variation as an instrumental variable (IV) for an exposure to estimate a causal effect. In principle (ie, under certain assumptions), this estimate is free from confounding, including reverse causation.^1^ It is still a relatively new technique and, due to advances in genetic research, it is becoming more popular and widely used.^2^ Large cohort studies, such as the UK Biobank (UKB) which contains genetic, health and lifestyle data for around half a million people, have made it relatively easy to perform powerful MR analyses quickly.^3^ Furthermore, platforms such as MR-BASE^4^ have made it quick and easy to conduct two-sample MR (ie, where the IV–exposure and IV–outcome associations come from different samples)^5^ with publicly available genome-wide analysis scan (GWAS) summary data. While reporting guidelines such as the Strengthening the Reporting of Observational Studies in Epidemiology (STROBE)-MR^6^ have recently been developed, it is not currently known whether MR studies across the whole field report their analyses appropriately and report them in enough detail for others to accurately replicate and interpret them.

Currently, while some tools for assessing the risk of bias in MR studies exist, none of these have been tested or validated for general use.^7^ Also, due to the extra information on genetic variants and the differing assumptions, tools for assessing bias in conventional public health research are not appropriate. A previous review on MR-Base studies found that 44% of studies provided sufficient detail on the first core assumption (ie, the genetic instrument is causally associated with the exposure), 31% on the second (ie, the genetic instrument shares no common cause with the outcome), 89% on the third (ie, the genetic instrument only has a causal effect on the outcome via the exposure) and 32% on assumptions of falsification tests.^8^ Another previous systematic review found that only 44% of MR studies discussed the plausibility of the core assumptions, and 14% gave insufficient detail of the statistical analysis.^9^ However, this review only looked at articles pre-2014, before both UKB became available and before MR became popular and widely used in epidemiology. Furthermore, while this review looked at a broad spread of MR methodologies, it only assessed the articles on these two points meaning its focus was rather narrow. Another review assessed the reporting quality of MR articles (up to 2017) on cancer outcomes and found around half the articles included (40%–69%) did not report subject characteristics, did not conduct power calculations, did not describe the core MR assumptions and did not exclude variants that failed certain quality control criteria.^10^ This review assessed more recent articles and assessed these articles in more detail but focused on the narrow topic of cancer MR studies.

The aim of our study is to assess whether published articles that conduct MR analyses using individual-level data from the UKB cohort use appropriate analyses and report enough details to allow accurate interpretation and replication and whether this varies across article type. The findings will highlight which specific characteristics of MR analyses are omitted. As MR is a rapidly expanding field, it is vital we make sure the research is being accurately conducted and reported so it can be adequately interpreted and replicated. This will lead to work in the field being more robust, which in turn will lead to a reduction in wasted resources and an increase in impact.

## Methods

The guidelines for reporting meta-epidemiological methodology research^11^ were used when writing this manuscript. We do not provide measures of interrater reliability as conflicts were often resolved by a consensus being reached between two or three reviewers. This study was preregistered on the Open Science Framework and the protocol can be found here https://doi.org/10.17605/OSF.IO/NWRDJ.

### Article search and eligibility criteria

We searched four databases (Web of Science, EMBASE, PubMed and PsycINFO) for articles, which contained “UK Biobank”, “UKB” or “UKBiobank” and “Mendelian randomisation” or “Mendelian randomization” on 15 July 2022 (online supplemental table S1).

10.1136/bmjebm-2022-112006.supp1 Supplementary data

We only included peer-reviewed, English-language (due to feasibility), original research articles, which were non-retracted and were open-access or fully accessible via our institution, which conducted MR analysis. We excluded:

- Articles where there were more than 10 independent exposures and/or outcomes (eg, phenome-wide association studies). This was due to feasibility.
- Articles which did not obtain a causal estimate of the exposure on the outcome.
- Articles which did not use solely individual-level data from UKB to obtain information on the IV–exposure and IV–outcome relationship when calculating the causal estimate, (ie, studies which pooled the data with other cohorts or conducted two-sample MR with publicly available summary level GWAS data).
- Articles for which eligibility was unclear.

This study includes articles which apply two-sample MR methods on UKB data either by utilising a split-sample approach or using the same UKB sample to estimate the IV–exposure and IV–outcome associations.

Article eligibility was assessed by one reviewer for title and abstract screening. For an initial batch of 64 papers (published before 4 November 2022 and found in the initial search which took place at the beginning of the project) full texts were screened by two reviewers independently, with a third reviewer resolving any conflicts when a consensus could not be reached. Articles were full text screened by a single reviewer in the second batch (articles found in the updated search conducted during the peer-review process, as requested by the editor).

### Data extraction

Data extraction was carried out by two independent reviewers for each article, with any conflicts being resolved by a third reviewer when a consensus could not be reached, in batch 1. Data were extracted by a single reviewer in batch 2. No reviewer reviewed their own article. Articles were reviewed using a 25-item checklist (See the Code Ocean repository at https://doi.org/10.24433/CO.4457049.v4 for full list^12^). On each item, the reviewer answered either ‘yes’, ‘partially’ or ‘no’, with ‘unclear’ or ‘NA’ also being allowed responses for specific items.

Both the STROBE-MR guidelines^6^ and the ‘Guidelines for performing Mendelian Randomisation investigations’^13^ were used to create the data extraction items. Each item is based on an item or items from the STROBE-MR while the ‘Guidelines for performing Mendelian Randomisation investigations’ were used to finalise the wording of each question to make sure it covers appropriate analytical practices as well as reporting practices. To measure how reporting quality differs across journals and article types, the 2021 Journal Impact Factor, journal word limit/recommendation, year of publication, whether the analysis was the primary analysis (with articles for which the MR analysis is the joint primary analysis coded as ‘partially’), open access status (with ‘free access’ articles being coded as ‘partially’) and whether the authors followed reporting guidelines (with the following of the STROBE-MR being coded as ‘yes’ and other less relevant reporting guidelines being coded as a ‘partially’), were also extracted.

First, a project protocol including initial data-extraction items was created. Then a pilot of ten articles in batch 1 was conducted to finalise the data-extraction items. During batch 1 data-extraction, the wording of some items was altered slightly to remove ambiguity in certain cases, while maintaining the intended meaning. We extracted information on the reporting of assumptions, design, variables of interest, the sample/data, the IV, MR estimator, the addressing of bias, the results and software and code.

### Statistical analysis

The percentage of articles which obtained a ‘yes’, ‘partially’ and ‘no’ (also ‘unclear’ where relevant) on each item of interest was calculated. Univariable linear regression was then used to investigate potential associations between the completeness of articles (average percentage across items for each article with ‘yes’ as 100%, ‘partially’ as 50% and ‘no’ or ‘unclear’ as 0%) and the year of publication, 2021 Journal Impact Factor (logged), word limit/recommendation (both number of words and whether a word limit/recommendation was present or not—articles from journals with page or character limits were large and were classed as having no word limit), whether the MR analysis was the primary analysis (answers of ‘yes’ were coded as 1, ‘partially’ as 0.5 an ‘no’ as 0), whether the article was open access (‘yes’ coded as 1 and ‘partially’ or ‘no’ coded as 0) and whether the articles followed reporting guidelines (‘yes’ and ‘partially’ coded as 1 and ‘no’ coded as 0). Due to limited variation in both open access statement and relevant reporting guideline use (as most articles were published before the creation of the STROBE-MR) we collapsed certain categories together. As we did not specify these variables would be handled this way in our preregistration, these analyses should be viewed as exploratory. The analysis for completeness on year was rerun adjusting for batch as a sensitivity analysis due to the fact that batch 2 articles were published after batch 1 articles but were also reviewed by a single reviewer rather than two and also reviewed at a later date (which could bias the association between article completeness and year of publication). All analyses were carried out in R V.4.1.0 and are available along with the data at https://doi.org/10.24433/CO.4457049.v4.^14^

## Results

**Figure 1:**
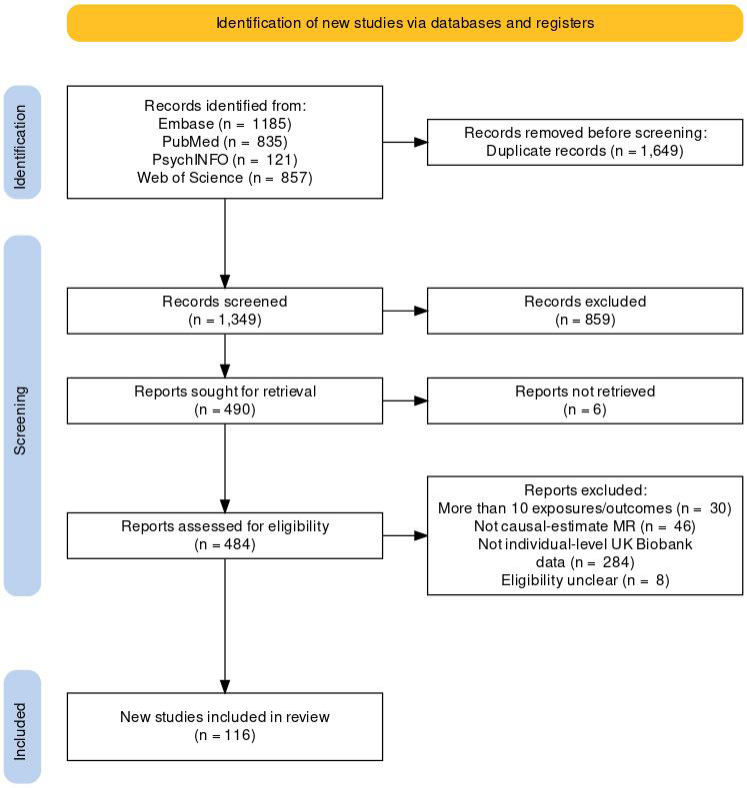
PRISMA style flow chart of article screening. MR, Mendelian randomisation.

**Figure 2:**
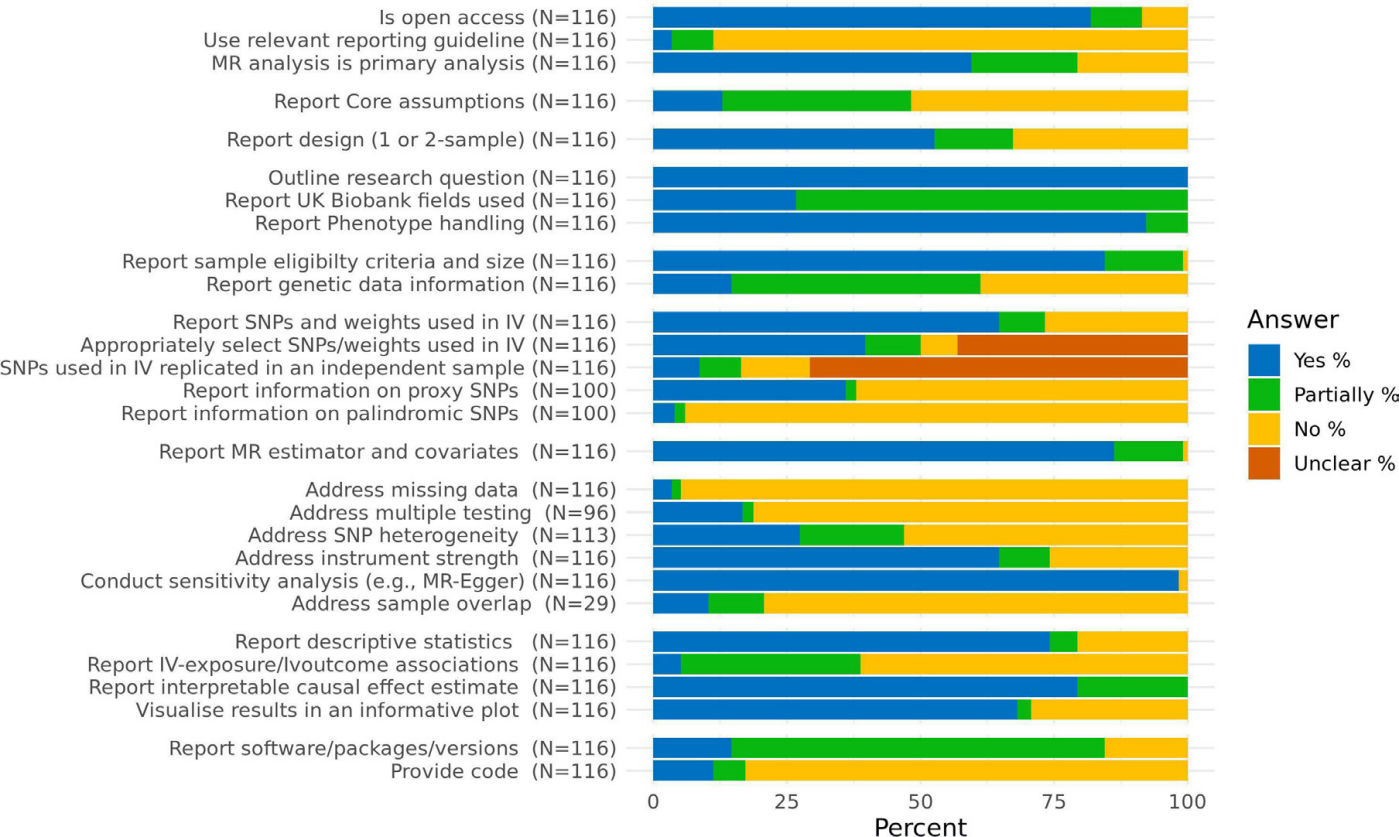
Percentages of each response for each article characteristic and data extraction item. Note: The number of articles for each item varies due to some items not being applicable for all articles. MR, Mendelian randomisation. SNP, single-nucleotide ploymorphism.

A total of 116 articles were included in the final sample (see figure 1 for a flow chart of article exclusion). The articles excluded at full text screening, the articles included in the review and the data extracted for each article can be seen at the Code Ocean repository (https://doi.org/10.24433/CO.4457049.v4). Overall, the mean article completeness was 55% (SD = 9%). Percentages for each item can be seen in figure 2 and at Code Ocean (https://doi.org/10.24433/CO.4457049.v4).

### Reporting the assumptions of MR

Of the 116 articles included in this review, only 13% (15) of articles completely reported the three core assumptions of MR, while 35% (41) partially reported them (ie, reported them incorrectly) and 52% (60) did not outline all three assumptions/did not outline them at all. While these assumptions have been previously detailed in a number of articles and reporting them it is not necessary for replication, they are important for the interpretation of the results and will not be common knowledge to those who do not conduct MR themselves. Often, they are reported wrong with the second assumption being reported as ‘no association between the IV and confounders of the exposure–outcome relationship’, rather than ‘the IV shares no common cause with the outcome’. The distinction between these assumptions is that the former is a specific form of pleiotropy that is already covered by the third assumption, whereas the latter covers important and otherwise unmentioned issues such as population stratification and dynastic effects.^15^

### Reporting the design

Articles regularly did not clearly report whether the study was a one-sample or two-sample MR design. While 53% (61) did report this accurately, 15% (17) only partially reported this (ie, implied its design in reference to it not being the alternative) and 33% (38) did not. This information is not only trivial to provide but helps to clearly communicate how the analysis was conducted (and therefore how it can be replicated) without having to infer this from more complicated details. It is also important to understanding the biases the results may be subject to and, thus, it is vital for the interpretation of the results.

### Reporting the variables of interest

As would be expected, 100% of articles completely reported what exposure outcome relationship was being assessed. However, only 27% (31) of articles completely reported which UKB phenotypes were used vs 73% (85) which partially reported this (ie, did not provide the UKB field IDs). UKB has many similar and closely related variables and without the IDs it is difficult to be certain which exact variable has been used by researchers. Reporting of field IDs can remove this ambiguity; 92% (107) of articles clearly reported how these variables were handled in enough detail to replicate the analysis and interpret the results, vs 8%^9^ which only partially reported this.

### Reporting the sample

For information on the eligibility criteria and subsample size, 84% (98) of articles completely reported this information while 15% (17) only partially reported this and 1% (1) did not. As sample size and exclusion criteria are vital to reporting in all fields of science this high rate is unsurprising. In contrast, only 15% (17) of articles completely reported information on the genetic data (ie, UKB microarrays, exclusion of variants and imputation information) while 47% (54) partially reported this information and 39% (45) didn’t report it at all. As these processes were mostly conducted by UKB centrally, most researchers may feel there is no need to report them. However, this information aids interpretation for those unfamiliar with UKB.

### Reporting IV information

Of the 116 articles included in the study, 65% (75) completely reported the genetic variants and weights used to construct the IV, while 9% (10) partially reported this and 27% (31) did not. 40% (46) reported that variants were identified in a different sample as that used in the analysis, or externally weighted, 10% (12) reported only that variants were identified in the same sample and unweighted or identified and weighted in a larger sample which includes the sample used in the MR analysis, 7% (8) reported that variants were identified and weighted from the same sample used in the MR analysis and 43% (50) did not report enough detail to assess this. Whether the variants used had been independently replicated had lower reporting quality across articles; 9% (10) of articles reported that variants were independently replicated, while 8% (9) reported they were partially replicated (eg, replicated in a partially overlapping sample), 13% (15) used unreplicated variants and 71% (82) did not report this. While using variants which were identified in the same sample, or which were not replicated can introduce bias, it is sometimes unavoidable. However, it is vital that this is outlined in the article as a possible source of bias and its potential impact should be discussed. For the 100 articles for which information on proxies and palindromic variants were relevant (ie, used variants for the IV which were not identified in solely UKB participants), 36% (36) of articles clearly reported proxy information (ie, whether all variants were present in UKB and, if not, whether these variants were excluded or proxied), 2% (2) partially reported this and 62% (62) did not. For the reporting of palindromic variants, 4% (4) clearly reported this if they were present and how they were handled if so, 2% (2) partially reported this and 94% (94) did not. Most researchers will feel that if palindromic or proxy variants were not present, they do not need to be mentioned. However, this creates ambiguity especially when many variants are being used. This information can always be included in to not disrupt the flow of the manuscript.

### Reporting analysis methods

Of the 116 articles, 86% (100) clearly reported the MR estimator used and the covariates adjusted for, while 13% (15) partially reported this and 1% (1) did not. While this rate of reporting is relatively high compared with the other items included in this review, this is fairly low considering how vital to replication and interpretation this information is.

### Addressing bias

Whether missing data could have biased the results was clearly addressed by 3% (4) of articles, while 2% (2) partially addressed this and 95% (110) did not (these articles may still have presented the percentages of missing data but did not comment on the impact of this issue). Of the 96 articles which conducted multiple testing, 17% (16) addressed this (ie, corrected for this bias or explained why no correction was needed), 2% (2) partially addressed this and 81% (78) did not. Many MR studies which investigate multiple exposures or outcomes will look at related, correlated variables and in these cases correcting for multiple testing will be too conservative. In these cases it may be preferable to not correct for this but this decision needs to be justified, which it often was not. For the 113 articles for which it was applicable (ie, more than one variant was used), 27% (31) addressed heterogeneity (ie, reported heterogeneity or used a method which excluded outliers) of the individual variant MR estimates, while 19% (22) partially did and 53% (60) did not. Reporting of assessments of instrument strength (ie, the F-statistic or the R^2^/variance explained) was better, with 65% (75) of articles completely reporting this, 9% (11) partially reporting this and 26% (30) not reporting this. Furthermore, the conducting and reporting of sensitivity analysis (any analysis to assess whether violations of assumptions are biasing the results) was common across articles, with 98% (114) completely reporting this and 2% (2) not. However, sensitivity analysis is a broad category of analyses, and this does not mean the analyses were conducted well or were the best suited sensitivity analyses for the study in question. Finally, of the 29 articles which used two-sample MR methods on one-sample data, only 10% (3) of articles addressed this potential source of bias, while 10% (3) partially addressed this and 79% (23) did not. Previous evidence suggests that sample overlap may not have large effects in terms of biasing the results for some two-sample MR methods,^16^ however, this should still be addressed by the authors.

### Reporting results

Descriptive statistics were completely reported by 74% (86), partially reported by 5% (8) and not reported by 21% (24) of articles. Reporting rates were far worse for the IV–exposure and IV–outcome associations which were completely reported by 5% (6), partially reported (ie, either only one of the two reported or reported for each individual variant) by 34% (39) and not reported by 61% (71) of articles. For reporting of the causal estimate reporting rates were (as would be expected) far better, with 79% (92) of articles completely reporting this and 21% (24) partially reporting this (ie, not making the units clear, not reporting an interpretable scale, or not giving a measure of uncertainty). Finally, 68% (79) of articles visualised the results in a figure, with 3% (3) only partially doing this (ie, the figure was not the best choice for visualisation or was difficult to interpret) and 29% (34) not visualising the results at all.

### Providing software and code

Of the articles, 15% (17) clearly reported the statistical programmes, packages and versions of each used for the statistical analysis, 70% (81) partially reported this (missing versions or packages) and 16% (18) did not. If complete code is provided then omissions of packages used and version numbers would matter little, however, only 11% (13) of articles provided complete code, 6% (7) provided partial code and 83% (96) did not provide any code. The provision of complete and readable code is vital to improving replicability and would cover most of the other items in this review.

### Effects of article characteristics

**Figure 3:**
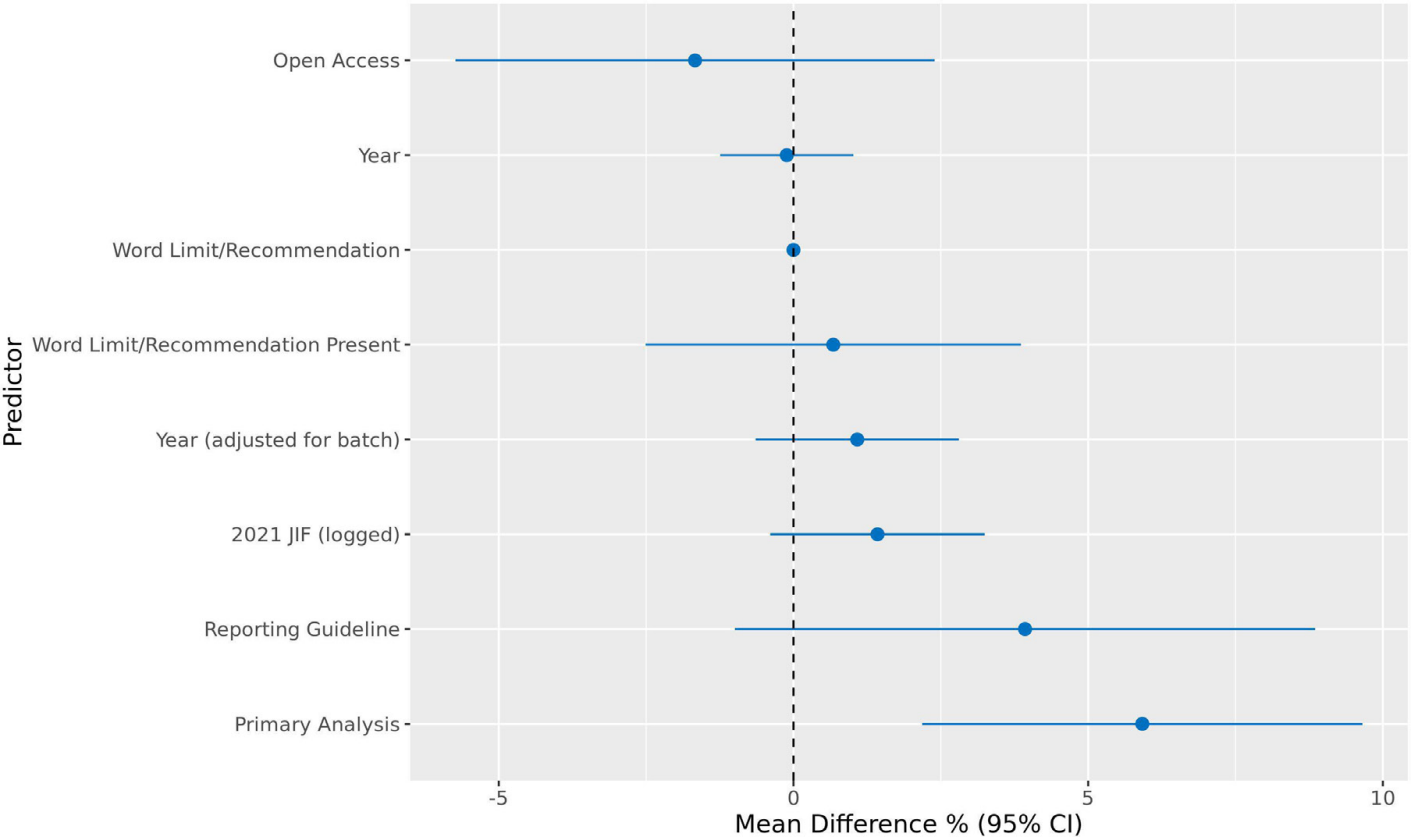
Regression results of article completeness percentage on article/journal characteristics. Note: N = 116 for all except word limit/recommendation where N = 68. Open access, reporting guideline and word limit/recommendation present are binary variables. Year, 2021 Journal Impact Factor (logged) and word limit/recommendation are continuous variables. For primary analysis, primary analysis = 1, joint-primary analysis = 0.5 and secondary analysis = 0. The association with each predictor was analysed in a univariable regression unless specified otherwise.

There was no clear evidence that 2021 Journal Impact Factor, word limit/recommendation (both word number or whether a limit/recommendation was present) or year of publication (unadjusted or adjusted for batch) predicted percentage of article completeness across items. There was evidence that primary analysis status (‘yes’ = 59% (69), ‘partially’ = 20% (23) and ‘no’ = 21% (24)) predicted an increase in completeness (mean difference in completeness percentage (95% CI) with primary analysis coded as 1, joint-primary coded as 0.5 and secondary analysis coded as 0 = 6% (2%, 10%)). While it might be expected that articles which did an eligible MR analysis as a joint primary analysis or secondary analysis would report this analysis less completely than those which conducted MR as the sole primary analysis, the ability to report methods and results in supplementary materials means researchers are always able to report analyses and results fully. This finding implies that researchers do not make proper use of supplementary materials and do not report all they should in these. Exploratory analyses for whether open access status (‘yes’ = 82% (95), ‘partially’ = 9% (11) and ‘no’ = 9% (10)) or the use of reporting guidelines (‘yes’ = 3% (4), ‘partially’ = 8% (9) and ‘no’ = 89% (103)) predicted article completeness, showed no clear evidence. Regression results can be seen in figure 3 and at the Code Ocean repository (https://doi.org/10.24433/CO.4457049.v4).

## Discussion

This study shows that the quality of analyses and reporting varied greatly across items and was worst for aspects relating to palindromic variants, variant replication, missing data, associations of the IV with the exposure/outcome and bias introduced by two-sample methods used on a single sample. Article completeness was not clearly predicted by 2021 Journal Impact Factor, word limit/recommendation or year of publication, but was predicted by primary analysis status. Exploratory analyses on whether open access status or the use of reporting guidelines predicted article completeness found no clear evidence of associations.

The results of this study roughly align with those of previous reviews. A previous study found that 44% of MR articles discussed the plausibility of the core assumptions^9^ and another found that 31%–89% of studies gave sufficient information on the validity of the core assumptions,^8^ while our study did not look at this specifically, we found that 48% outlined the core assumptions (although mostly incorrectly), 27% completely addressed variant heterogeneity (relevant to the third core assumption), and 65% completely addressed the strength of the IV (ie, the first assumption). However, the previous studies did not investigate the reporting of variant heterogeneity like we did, and one found lower reporting of instrument strength than us (30%–34%). This previous study also identified that 14% of studies gave insufficient detail of the statistical analysis (ie, information to accurately replicate including the CIs of the results), while we found that 14% did not give complete information about the MR estimator and covariates and 21% did not provide the causal estimate on an interpretable scale with measures of uncertainty. The other study (on MR-BASE analyses) showed similarly high rates of defining the exposure/outcome relationship being tested and defining the variables of interest (92%–97%), similarly middling rates of describing how variants were identified (68%) and similarly low rates of describing data harmonisation (14%–25%) and providing code (8%). They also found no evidence that reporting quality changed over time, supporting our findings. However, they found lower rates of reporting the causal estimate on an interpretable scale than we did (52% vs 79%). This study also reviewed articles with more than 10 exposures/outcomes (but stratify the results based on this) while we did not, and only rated articles on whether they did or did not provide information, while we also recorded whether information was partially provided. Another review (on oncology MR studies) found that 49% of articles reported subject characteristics and 48% did not describe the core MR assumptions. We found higher rates of reporting of subject characteristics (84%) and similar rates of not reporting the core assumptions (52%) in a broader sample of MR articles. It is important to note that no articles from the current review were included in the previous reviews.

Strengths of this study are the comprehensiveness of the review items and that articles from the first batch (55% of the overall sample) were full text screened and reviewed by two reviewers with a third resolving conflicts, reducing the impact of bias and human error. This also allowed for the data extraction items to be refined and a consensus to be agreed on how each item was answered between the reviewers. One limitation, however, is that due to time restraints, articles from the second batch were screened and reviewed by the primary author only. As this was conducted after the screening and reviewing of batch 1 was completed, and thus a consensus on how each item should be answered had already been agreed on by the authors, this is unlikely to have a large effect on the data other than an increased risk of error. However, the mean article completeness of batch 2 was similar to the mean for each second reviewer and all were within one SD of each other. A further limitation is that overall article completeness is an arbitrary measure which assumes each item is equally important. While that is untrue, any weighting applied to this measure would be subjective and therefore just as arbitrary, and as the measure is only intended to identify predictors of article quality, we believe it is suitable to construct it in this manner. Also, while this review includes a large number of MR articles across all fields, it focuses on a very specific subset: those which used individual-level UKB data only to obtain a causal estimate for less than 10 exposures or outcomes. To include articles outside of this subset would have made the review too large but it is possible that article completeness differs drastically outside this subset. Further, several methods articles are included in the review. Many would argue if an analysis is just being done to demonstrate a method it does not need to be reported in detail. We acknowledge there is some merit to this argument but feel best practice is always to report any analyses in a reproducible manner. Finally, several items could have been coded in more detail if the scope of the project was not already so large (ie, the quality of sensitivity analysis or code provided could have been extracted). The replicability of articles would also be better assessed by carrying out full replication attempts. These are two areas which should be followed up in future research, if only on a small subsample of articles. It would also be worthwhile to investigate whether the adoption of the STROBE-MR, preregistration,^17^ sharing materials^14^ or peer-review^18^ improves article completeness, however, more time needs to pass from its creation for the former to be feasibly investigated.

## Conclusions

The findings of this study highlight areas of poor conduct and reporting in MR research which need to be improved to increase replicability and impact. Only primary analysis status clearly predicted article completeness implying that researchers do not sufficiently use supplementary materials to report secondary analyses and results. Increased analysis and reporting quality in the field is vital for improving our ability to replicate and accurately interpret findings, increasing the impact of research and making better use of public money. Future research should focus on the quality of certain aspects such as code or sensitivity analysis, as well as attempting direct replications, and should investigate the impact of the STROBE-MR specifically once it is more widely used.

## Data Availability

Data are available in a public, open access repository. The data are available via the University of Bristol online data repository athttps://doi.org/10.5523/bris.2voc5xy13adqs2rimeateez14yand at https://codeocean.com/capsule/1654662/tree/v1.
