# Supplementary material for: Reporting and methodological quality of studies that use Mendelian randomisation in UK Biobank: a meta-epidemiological study
Source: BMJ Evid Based Med. 2022 Dec 8;28(2):103–10. doi: 10.1136/bmjebm-2022-112006 (PMC10086297; doi:10.1136/bmjebm-2022-112006)
Supplement: Supplementary data [file bmjebm-2022-112006supp001.pdf]

Reporting and methodological quality of studies that use Mendelian randomisation in UK Biobank: a meta-epidemiologic study

Mark Gibson et al.

Supplementary Table S1

Supplementary Table S1.

Details of search conducted in four databases on 15/07/2022.

Database (Vendor)	Search strategy syntax	Search syntax meaning	URL
Web of Science Core Collection	(TS=("UK Biobank" OR UKB OR UKBiobank) OR ALL=("UK Biobank Resource" OR "UKB Resource")) AND (TS=("Mendelian randomisation" OR "Mendelian randomization") OR ALL=("Mendelian randomisation" OR "Mendelian randomization"))	ALL = all fields<br>TS = title, abstract, author, keywords, Keywords Plus® [1]	https://apps.webofknowledge.com/WOS_AdvancedSearch_input.do?SID=F2upfRKXSkaPxfXaEDL&product=WOS&search_mode=AdvancedSearch
PubMed	((((("Mendelian randomisation") OR "Mendelian randomization") OR "Mendelian randomisation"[TIAB]) OR "Mendelian randomization"[TIAB]) OR "Mendelian Randomization Analysis"[Mesh]) AND ((((("UK Biobank Resource") OR "UKB Resource") OR UKB[TIAB]) OR UKBiobank[TIAB]) OR "UK Biobank"[TIAB])	No syntax = all fields<br>TIAB = citation's title, collection title, abstract, other abstract and author keywords<br>Mesh = Mesh term [2]	https://pubmed.ncbi.nlm.nih.gov/
EMBASE (Ovid)	(UK Biobank resource or UKB Resource).af. or UK Biobank.ab. or UKB.ab. or UKBiobank.ab. or UK Biobank.ti. or UKB.ti. or UKBiobank.ti. or UK Biobank.kw. or UKB.kw. or UK Biobank.kw.	af = all fields<br>ab = abstract<br>ti = title<br>kw = author keywords[3]	https://ovidsp.dc1.ovid.com/ovid-a/ovidweb.cgi
	(Mendelian randomisation or Mendelian randomization).af. or Mendelian randomisation.ab. or Mendelian randomization.ab. or Mendelian randomisation.ti. or Mendelian randomization.ti. or Mendelian randomisation.kw. or Mendelian randomization.kw.		
	Combine 1 and 2		
PsycINFO (Ovid)	("UK Biobank resource" or "UKB Resource").af. or ("UK Biobank" or UKB or UKBiobank).ab. or ("UK Biobank" or UKB or UKBiobank).ti. or ("UK Biobank" or UKB or UKBiobank).id.	af = all fields<br>ab = abstract<br>ti = title<br>id = key concepts [4]	https://ovidsp.dc1.ovid.com/ovid-a/ovidweb.cgi
	("Mendelian randomisation" or "Mendelian randomization").af. or ("Mendelian randomisation" or "Mendelian randomization").ab. or ("Mendelian randomisation" or "Mendelian randomization").ti. or ("Mendelian randomisation" or "Mendelian randomization").id.		
	Combine 1 and 2		

[1] http://images.webofknowledge.com/images/help/WOS/hs_advanced_fieldtags.html
[2] https://www.ncbi.nlm.nih.gov/books/NBK3827/#pubmedhelp.TitleAbstract_TIAB
[3] http://ospguides.ovid.com/OSPguides/embase.htm#kw
[4] https://ospguides.ovid.com/OSPguides/psycdb.htm
